# Transcriptome sequencing reveals the key genes associated with hair follicle development in Qianhua Mutton Merino

**DOI:** 10.3389/fvets.2025.1699868

**Published:** 2025-12-04

**Authors:** Zhiyun Qin, Xinming Sun, Limin Sun, Meng Yu, Huaizhi Jiang

**Affiliations:** 1 Key Laboratory of Evaluation and Utilization of Livestock and Poultry Resources (Sheep), Ministry of Agriculture and Rural Affairs, Jilin Agricultural University, Changchun, China; 2 Northeast Agricultural Research Center of China, Jilin Academy of Agricultural Sciences, Changchun, China; 3 Liaoning Anyou Biotechnology Co., ltd., Shenyang, Liaoning, China

**Keywords:** Qianhua mutton Merino sheep, hair follicle development, DEGs, KRT, S/P

## Abstract

**Background:**

The aim is to compare the morphological changes and gene expression profiles of hair follicles in newborn and one-year-old Qianhua Mutton Merino sheep, explore the changes in gene expression and related regulatory pathways during the process from incomplete development to full development of skin hair follicles, and identify the key genes influencing hair follicle development and maturation. This study holds significant theoretical importance for revealing the molecular regulatory mechanism of hair follicle development in Qianhua Mutton Merino sheep.

**Methods:**

A total of five newborn (1-day-old) and five yearling (12-month-old) Qianhua Mutton Merino sheep were selected for this study. Skin tissue samples were collected for histological analysis using HE staining. Transcriptome sequencing was performed on the Illumina NovaSeq 6000 platform, and differentially expressed gene (DEG) analysis was conducted using the R package degeR. GO and KEGG pathway enrichment analyses were performed on the DEGs to identify genes associated with hair follicle development. Ten DEGs were randomly selected for the validation of the transcriptome sequencing results using realtime quantitative PCR. The *KRT27* gene was further validated through immuno-histochemistry and quantitative PCR.

**Results:**

The results from HE staining indicated that with an increase in the age of the Qianhua Mutton Merino sheep, the density of both primary and secondary hair follicles significantly decreased (*p* < 0.05), while the S/P ratio exhibited a remarkable increase (*p* < 0.05). The transcriptome sequencing results identified a total of 1,637 differentially expressed genes, of which 1,134 were upregulated and 503 were downregulated. GO and KEGG enrichment analyses of DEGs related to hair follicle development revealed that these genes were enriched in signaling pathways associated with hair follicle development, such as the PI3K-Akt, MAPK, and Estrogen signaling pathways. The results of real-time quantitative PCR were consistent with the transcriptome sequencing results. The *KRT27* gene was expressed in the inner root sheath region of hair follicles, with a significant upregulation observed at 12 months of age (*p* < 0.05).

**Conclusion:**

In conclusion, this study identified the *KRT27*, *IGF-2*, *FGF21*, *VEGF-D*, *KRT25,* and *KRT26* genes as important candidate genes in the development of hair follicles in Qianhua Mutton Merino sheep through transcriptome sequencing analysis, providing a theoretical basis for further revealing the molecular mechanisms underlying the formation of wool traits in Qianhua Mutton Merino sheep.

## 1 Introduction

The hair follicles of sheep are the “source” of their fleece and the only organs that undergo periodic changes. Based on their morphological structure and developmental timeline, hair follicles can be categorized into primary follicles (PFs), which produce myelinated hairs, and secondary follicles (SFs), which produce unmyelinated hairs. Several secondary hair follicles form clustered follicle groups of around one to seven primary hair follicles. Therefore, the characteristics and tissue structure of sheep hair follicles determine the quality and yield of wool (1). As hair follicles rely on periodic cyclical growth to achieve fleece renewal, their cyclical changes dictate the growth and shedding of the fleece. The periodic growth and development of hair follicles are influenced by various factors and regulated by a series of signaling molecules. With the widespread application of second-generation high-throughput sequencing technology, it has been discovered that multiple signaling pathways, including Wnt (2), TGF-*β* (3, 4), PI3K-Art (5, 6), Notch (7), MAPK (8, 9), and BMP (10), play crucial regulatory roles in the morphological development of hair follicles. The Qianhua Mutton Merino sheep is a dual-purpose breed developed in China in 2018, characterized by its large body, rapid daily weight gain, high meat production performance, and excellent meat quality. Additionally, it primarily produces homogeneous fine wool with a count of 66 (11). Although extensive research exists on sheep hair follicle development, the signaling pathways governing this process exhibit species and breed specificity. This prior knowledge provides a crucial foundation for exploring the pathways and key genes in Qianhua Mutton Merino sheep. Previous studies have shown that the number of primary hair follicles in Qianhua Mutton Merino does not change significantly after rebirth. Newborn (1 day old) Qianhua Mutton Merino are in the young stage, and their hair follicles are in the process of growth and development, not yet fully mature. However, yearling (12 months old) Qianhua Mutton Merino use have fully developed hair follicles. Studying the skin hair follicles of newborn and one-year-old Qianhua Mutton Merino is helpful for exploring the complete development of hair follicles from immaturity to maturity. As Qianhua Mutton Merino sheep are a dual-purpose breed for both meat and wool, the two critical time points for follicle development are at birth and 1 year of age. The specific genes that play a central role during these two developmental stages, as well as the expression dynamics of these genes, require further in-depth study. In order to investigate the key genes involved in the development of hair follicles in Qianhua Mutton Merino sheep at birth and 1 year of age, histological and transcriptomic analyses were conducted on the skin tissues of newborn and one-year-old Qianhua Mutton Merino sheep. Differential genes at different develop-mental stages were screened, and their related functions were analyzed, providing a theoretical basis for further elucidating the molecular mechanisms underlying the formation of wool traits.

## 2 Materials and methods

### 2.1 Laboratory animals

Samples were collected from the core breeding group of Qian’an Zhihua Sheep Breeding Co., Ltd. (National Core Breeding Farm for Sheep). Skin samples were taken from the left scapular posterior edge of 5 newborn (M1) and 1-year-old (M12) ewes, and the sampling area measured 2cm × 2cm. The samples were divided into two parts: one part was preserved in 4% paraformaldehyde fixation solution, while the other part was transported back to the laboratory in liquid nitrogen and stored at −80 °C for future use.

### 2.2 Reagents and instruments

The following were obtained: anhydrous ethanol (Tianjin Chemical Reagent Co., Ltd.), paraformaldehyde (Tianjin No.3 Chemical Reagent Factory), Trizol (Invitrogen), neutral gum (Tianjin No.3 Chemical Reagent Factory), Tris-EDTA (Saiwen Innovation (Beijing) Biotechnology Co., Ltd.), a biological tissue embedding machine (Jinhua Yidi Medical Equipment Co., Ltd., YD-6D), a Rotary Microtome (Leica Instruments AG, Germany, Leica RM2235), a biological tissue baking machine (Jinhua Yidi Medical Equipment Co., Ltd., YD-AB), and a biological microscope (Leica Instruments AG, Germany, Leica DM 1000).

### 2.3 Paraffin-embedded skin tissue sections were used for hematoxylin and eosin (HE) staining

Skin samples were fixed with formaldehyde and embedded in paraffin, and continuous skin sections were prepared with a thickness of 4 μm. Hematoxylin and eosin staining was performed, followed by resin embedding to observe the structural organization of skin follicle tissues (12). Skin tissue exhibits a phenomenon in which it contracts in tissue fluid; therefore, it is necessary to correct the skin contraction rate. The skin contraction rate = the stained skin area / *in vivo* sample skin area. The skin contraction rates at birth and at 12 months of age were 0.61 and 0.78, respectively. All the skin data presented were adjusted for skin contraction rates. In the continuous sections obtained, transverse sections near the primary hair follicle sebaceous glands were selected to observe the number of primary and secondary hair follicles in each field of vision and calculate the follicular density and S/P. During measurement, the microscope was set to 100 times magnification, and the field-of-vision area was 2.9225 mm^2^.

### 2.4 RNA extraction, library construction, and sequencing

Total RNA was extracted, isolated, and purified from skin tissue samples using TRIzol. Quality control was performed using NanoDrop ND-1000, Bioanalyzer 2100, and agarose gel electrophoresis, ensuring that the concentration was > 50 ng/μL, the RIN value was > 7.0, the OD260/280 was > 1.8, and total RNA was > 1 μg before proceeding to downstream experiments. PolyA tail mRNA was captured through two rounds of purification using oligo(dT) magnetic beads, followed by fragmentation using a high-temperature magnesium ion fragmentation kit. cDNA was synthesized via reverse transcription, and subsequent steps included double-strand synthesis, end processing, adapter ligation, magnetic bead selection, and purification. The UDG enzyme was used to digest the double strands, and PCR amplification was performed to construct a library of 300 bp ± 50 bp. Finally, PE150 paired-end sequencing was conducted using the Illumina Novaseq™ 6000.

### 2.5 Bioinformatics analysis

The differentially expressed mRNAs were selected using thresholds of a fold change > 2 or < 0.5 and a parametric F-test comparing nested linear models (*p* value < 0.05) via the R package edgeR.^1^ We performed STRING protein–protein interaction analysis on differentially expressed mRNA to predict the protein interaction relationships of DEGs using the STRING database.^2^ The genes with the parameter of false discovery rate (FDR) below 0.05 and absolute fold change > 2were considered differentially expressed genes. Finally using DAVID software^3^ to GO and KEGG gene enrichment analysis. This analysis aimed to explore the interaction relationships of genes related to hair follicle development.

### 2.6 Real-time fluorescence quantitative PCR verification

Total RNA was extracted and reverse-transcribed into cDNA with a total reaction volume of 20 μL: nuclease-free water up to 20 μL, 4 μL of 5 × RT Buffer, 1 μL of RT Enzyme Mix, 1 μL of Primer Mix, and 2 μg of RNA. The reaction was denatured at 65 °C for 5 min, followed by a reverse transcription reaction at 37 °C for 15 min and, finally, treatment at 98 °C for 5 min. Based on the mRNA sequences of the sheep, *KRT27*, *CPT1A*, *MT3*, *MRC1*, *MYLPF*, *KRT25*, *KRT71*, *FCGR2B*, *IL18*, *HSD11B1*, and *GSTA1*, obtained from the NCBI database, primers for the cloning and amplification of the CDS region were designed using Primer 5.0 software. These primers were synthesized by GeneChem Co., Ltd. (Shanghai, China), and the primer sequences are shown in Table 1. Using cDNA as a template, the reaction system was set as follows 20μL: 6.4μL of distilled water; 10μL of SYBR Green Realtime PCR Master Mix; 0.8μL of primers; and 2μL of cDNA. The PCR reaction program consisted of an initial denaturation at 94 °C for 3 min, followed by 40 cycles of denaturation at 94 °C for 15 s and annealing at 60 °C for 30 s. Finally, an extension step was performed at 72 °C for 20 s, with a gradual increase from 65 °C to 95 °C over 5 s to obtain the melting curve. All samples were measured in triplicate. The resulting data were normalized using *β*-actin and calculated using the 2-ΔΔCt method.

**Table 1:** The primer information used in this investigation.

Gene	Primer Sequence 5′ to 3′	Expected product size
*KRT27*	F: GGGACTGGGCGGAGGAAGTG	108 bp
	R: GCCAGGCGGTCATTGAGGTTC	
*CPT1A*	F: CGGTTGCTGATGACGGCTATGG	114 bp
	R: TCCCGAAGCGATGCGAGTCC	
*MT3*	F: TGCACCTGCTCCGACTCCTG	150 bp
	R: GCAGCCGCACTTCTTCTCCTC	
*MRC1*	F: AGGGATGCGGGAAAGTGGATGG	87 bp
	R: ACTCGGCAAGGAAGGGTCAGG	
*MYLPF*	F: AGAGAAGGGCAGCAGCAGAGG	148 bp
	R: GCAAAGGTGTCCCGCAGGTC	
*KRT25*	F: CAACGCTGACCTGGAGCAGAAG	150 bp
	R: TTGGCATTGCTGGTGGTGGAAG	
*KRT71*	F: AGCTGCTGGAGAGTGAGGAGTG	81 bp
	R: CGCTGGTGCTGCTGATGATGG	
*KCGR2B*	F: AGCCTGCGTGGATCAATGTGC	86 bp
	R: CGTGGTGAGGTTGCCTGCATAG	
*IL18*	F: TGGCGAAGACCTGGAATCAGATC	96 bp
	R: CCCTGGCTAATGAAGAGAACTTGG	
*HSD11B1*	F: GCCAGCAAGGGAATCGGAAGAG	116 bp
	R: TCCAGGCAGCGGGATACCAC	
*GSTA1*	F: GACGCCAAGCTGACACCAATCC	100bp
	R: TGCCCACCAGGTAGTCTTGTCC	

### 2.7 Immunohistochemistry

For the immunohistochemical staining of 5μm thick tissue sections, the sections were deparaffinized using xylene and washed with PBS four times. The sections were stained according to the immunostaining reagents, with the *KRT27* antibody (dilution 1:500) incubated at 4 °C for 12 h, followed by three washes with PBS. The samples were counterstained with hematoxylin (Merck, Darmstadt, Germany) for 30 s and immediately washed with water. Finally, the samples were mounted with neutral resin and covered with a coverslip, and the staining was observed under an optical microscope.

### 2.8 Data analysis and statistics

An analysis of variance (One-Way ANOVA) for differences among different groups was conducted using SPSS 22.0 software. Graphs were generated using GraphPad Prism 9.5 software. The results are represented as the mean ± standard error, with *p* < 0.05 indicating a significant difference.

## 3 Results

### 3.1 The morphological structure of hair follicle tissues in Qianhua Mutton Merino sheep at different ages

**Figure 1:**
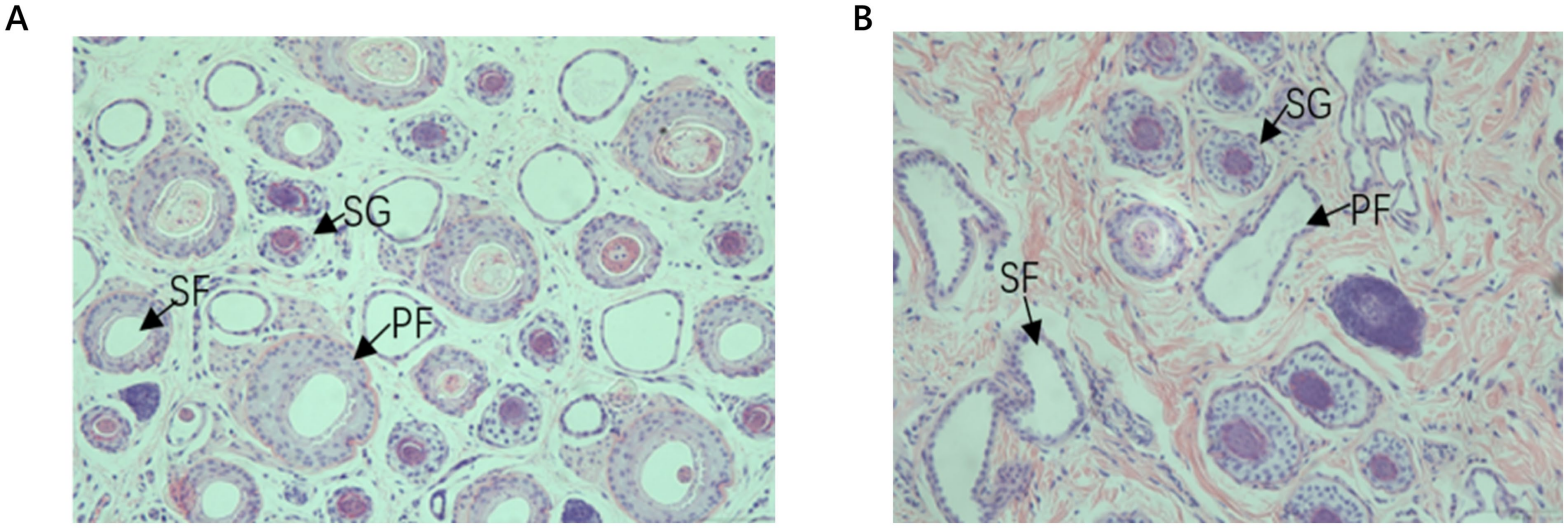
**(A)** 100 × the structure of newborn skin hair follicles; **(B)** 100 × the structure of 12-year-old skin hair follicles. PF, primary follicle; SF, secondary follicle; SG, sebaceous gland.

Through HE staining, we made morphological observations of the skin follicle structure in newborn (M1) and one-year-old (M12) Qianhua Mutton Merino sheep (Figures 1A,B). Structures such as primary follicles (PFs), secondary follicles (SFs), and sebaceous glands (SGs) were observed in both periods. The diameter of primary hair follicles is larger than that of secondary hair follicles, and they are surrounded by sebaceous and sweat gland structures. In the skin tissue of newborn Qianhua Mutton Merino sheep, the number of primary hair follicles is greater than that of secondary hair follicles, and at this time, secondary hair follicles are not densely distributed around primary hair follicles. The hair follicles of newborn Qianhua Mutton Merino sheep for meat use are still in the developmental stage, not fully mature, and have not yet entered the cyclical development of hair follicles.

In the skin tissue of the one-year-old Qianhua Mutton Merino sheep, the number of secondary hair follicles is greater than that of primary hair follicles, and they are distributed more densely, forming a hair follicle cluster. In this study, the skin samples of one-year-old Qianhua Mutton Merino sheep were collected in March. At this time, the hair follicles had undergone a complete hair follicle cycle and were in the resting phase. The Skin structure parameters of Qianhua Mutton Merino sheepin newborn and one-year-old sheep is presented in Table 2. The number of primary and secondary follicles in Qianhua Mutton Merino sheep shows a gradual decrease with increasing age. There were no significant differences (*p* > 0.05) in the diameter, depth of the primary hair follicles of Qianhua Mutton Merino sheep at birth and at one - year - old. However, significant differences (*p* < 0.05) were observed in the diameter and depth of the secondary hair follicles. This indicates that the development of primary hair follicles had been completed at birth, while the secondary hair follicles were still in the developmental stage. In addition, the difference in the diameter of wool fibers grown from secondary hair follicles (*p* < 0.05) in the fleece was also consistent with these findings. At birth, the density of PF and SF is significantly higher than that at 1 year of age (*p* < 0.05). However, at 1 year of age, the S/P ratio is significantly higher than that during the newborn period (*p* < 0.05).

**Table 2:** Skin structure parameters of Qianhua Mutton Merino sheep.

Project	M1	M12
PF /mm^2^	8.15 ± 1.04^a^	4.38 ± 0.21^b^
PF diameter/μm	90.62 ± 7.13	91.68 ± 7.24
PF depth /μm	865.09 ± 42.53	889.64 ± 61.63
SF /mm^2^	61.81 ± 3.45^a^	55.31 ± 5.21^b^
SF diameter/μm	80.61 ± 7.07^b^	85.65 ± 6.45^a^
SF depth /μm	702.05 ± 31.41^b^	846.45 ± 51.62^a^
WFD/μm	20.65 ± 1.42^b^	21.61 ± 1.09^a^
S/P	7.58 ± 0.64^b^	12.64 ± 1.02^a^

Different letters in the same row indicate significant differences (*p*﹤0.05), and the same letters indicate non-significant differences (*p*﹥0.05).

### 3.2 Analysis of transcriptome sequencing data obtained from Qianhua Mutton Merino sheep skin tissues at different ages

Ten skin tissue samples from Qianhua Mutton Merino sheep at different ages were subjected to library construction and sequencing. After preprocessing and filtering the raw sequencing data, we obtained high-quality effective reads. The total raw read counts ranged from 36,147,820 to 48,395,978 bp, with a base count between 5.42G and 7.26G. After filtering, the effective read counts were between 34,809,696 and 45,987,934 bp, with a base count ranging from 5.22G to 6.90G. The proportion of effective reads exceeded 95% (95.02–97.12%), retaining a large number of high-quality reads. The Q20 (base accuracy ≥ 99%) and Q30 (base accuracy ≥ 99.9%) proportions were in the range of 99.72–99.97% and 97.40–98.12%, respectively, indicating extremely high data accuracy. The GC content ranged from 47 to 50.5%, distributed evenly and in accordance with species characteristics. These metrics demonstrate that the quality of the sequencing data is excellent, providing a reliable foundation for subsequent analyses.

### 3.3 Sequencing data filtering and reference genome alignment

**Figure 2:**
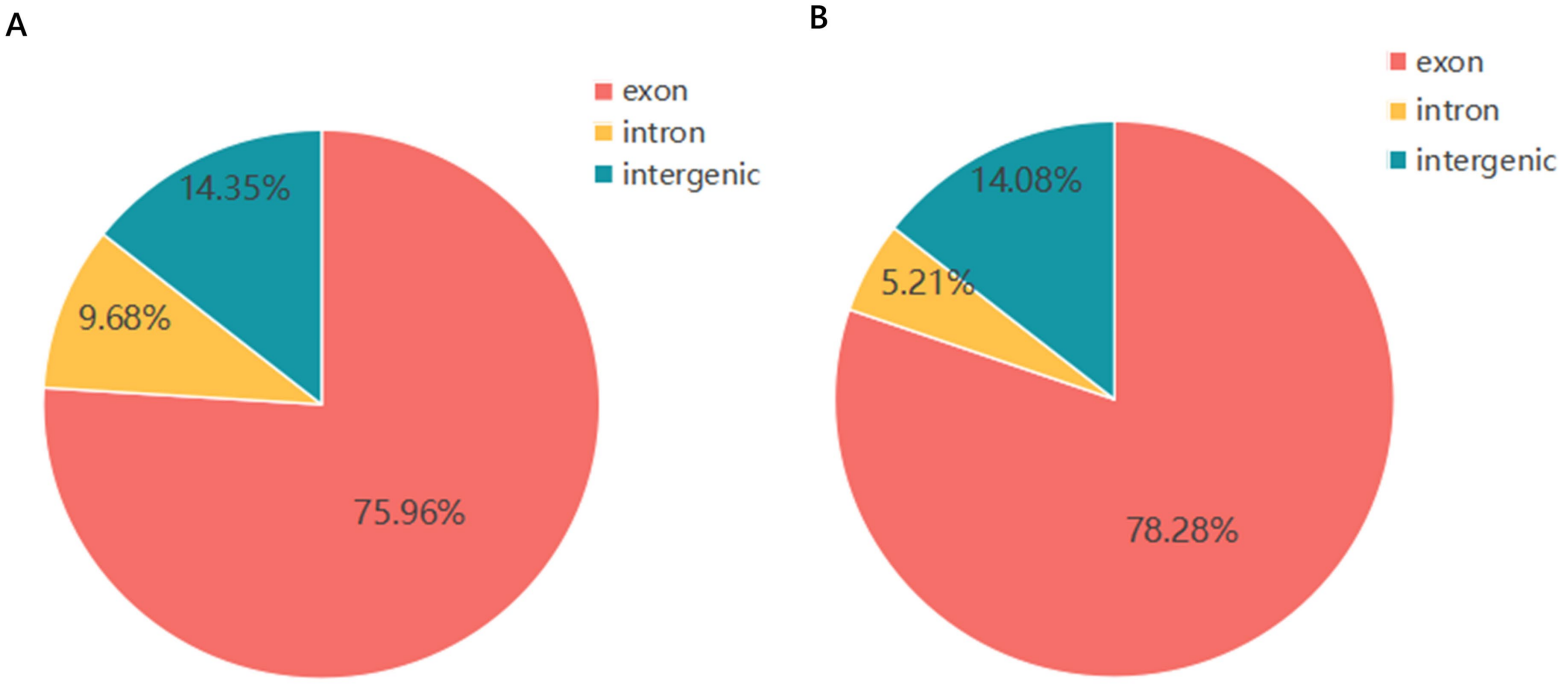
**(A)** Comparison of the regional distribution of group M1 and that of the reference genome; **(B)** comparison of the regional distribution of group M12 and that of the reference genome.

The distribution of reads compared to the reference genome was primarily concentrated in the exon, intron, and intergenic regions. In all the samples, the vast majority of reads were distributed in the exon regions, accounting for between 75.96 and 78.28% (Figures 2A,B), indicating that the sequencing data mainly originate from coding regions, a finding consistent with the expectations of mRNA sequencing. The next largest proportion was found in the intron regions, ranging from 9.68 to 14.08%, which may reflect unspliced precursor mRNA or other transcriptional fragments derived from introns. The proportion of reads in the intergenic regions was relatively low, ranging from 5.21 to 14.35%, a finding that may be due to non-coding RNA or background noise. Overall, the proportion of reads in the exon regions across all samples was relatively consistent, validating the reliability of the sequencing data and the accuracy of the reference genome alignment, thereby providing foundational data support for subsequent expression quantification and functional analysis.

### 3.4 Differentially expressed gene analysis

**Figure 3:**
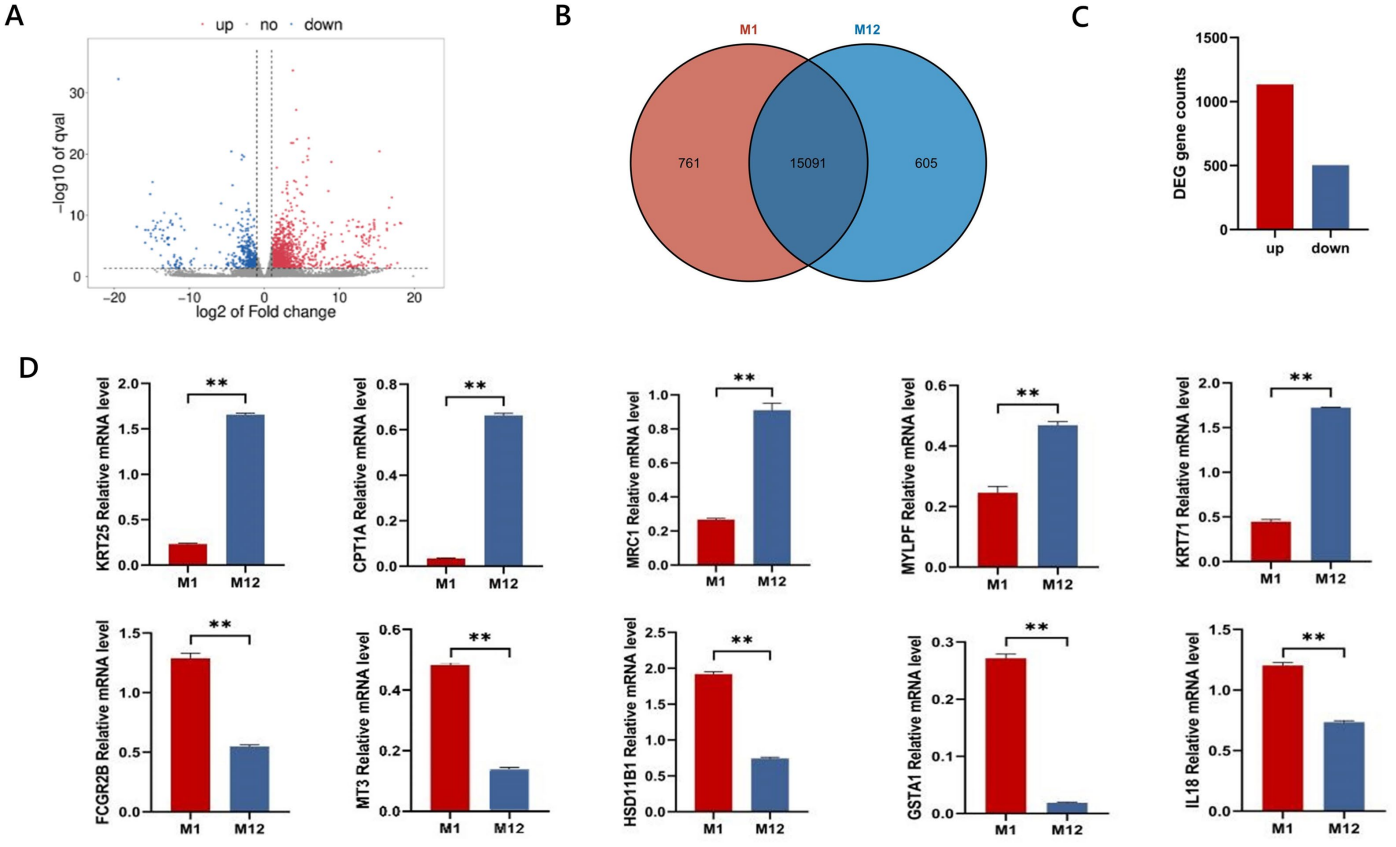
**(A)** Volcano plot of differential mRNA; **(B)** Venn diagram of genes; **(C)** downregulation and upregulation of differential genes; **(D)** RT-qPCR validation.** indicates significance at *p* < 0.01.

To screen for candidate genes related to hair follicle development at different stages in Qianhua Mutton Merino sheep, we compared two critical time points: newborn (M1) and 1 year old (M12). A total of 15,852 genes were identified in the M1 group, while 15,696 genes were identified in the M12 group, with a total of 15,091 common genes (Figure 3B). Using thresholds of a fold change > 2 or < 0.5 and a *p* value < 0.05, we detected 1,637 DEGs, including *CCL21*, *TPSB2*, *GSTA1*, *MRC1*, *ND1*, *MYLP*, *IL18*, *ND4L*, *GHRHR*, *ITGAM*, *LEPR*, *MMP2*, *ANKRD1*, *PLIN5*, and *DIRAS3*. Of these, 1,134 were upregulated and 503 were downregulated, with the number of upregulated DEGs significantly exceeding the number of downregulated DEGs (Figures 3A,C). Ten genes were randomly selected from the DEGs for RT-qPCR validation (Figure 3D). The expression levels of *CPT1A*, *KRT25*, *KRT71*, *MRC1*, and *MYLPF* were significantly upregulated in the M12 group (*p* < 0.01), while *GSTA1*, *FCGR2B*, *HSD11B1*, *IL18*, and *MT3* showed significantly downregulated expression levels in the M12 group (*p* < 0.01). The results of the differential gene RT-qPCR validation were largely consistent with the expression trends observed in the transcriptome sequencing analysis, thereby confirming the reliability of the data obtained from transcriptome sequencing.

### 3.5 KEGG pathway enrichment and GO functional annotation analysis for DEGs

**Figure 4:**
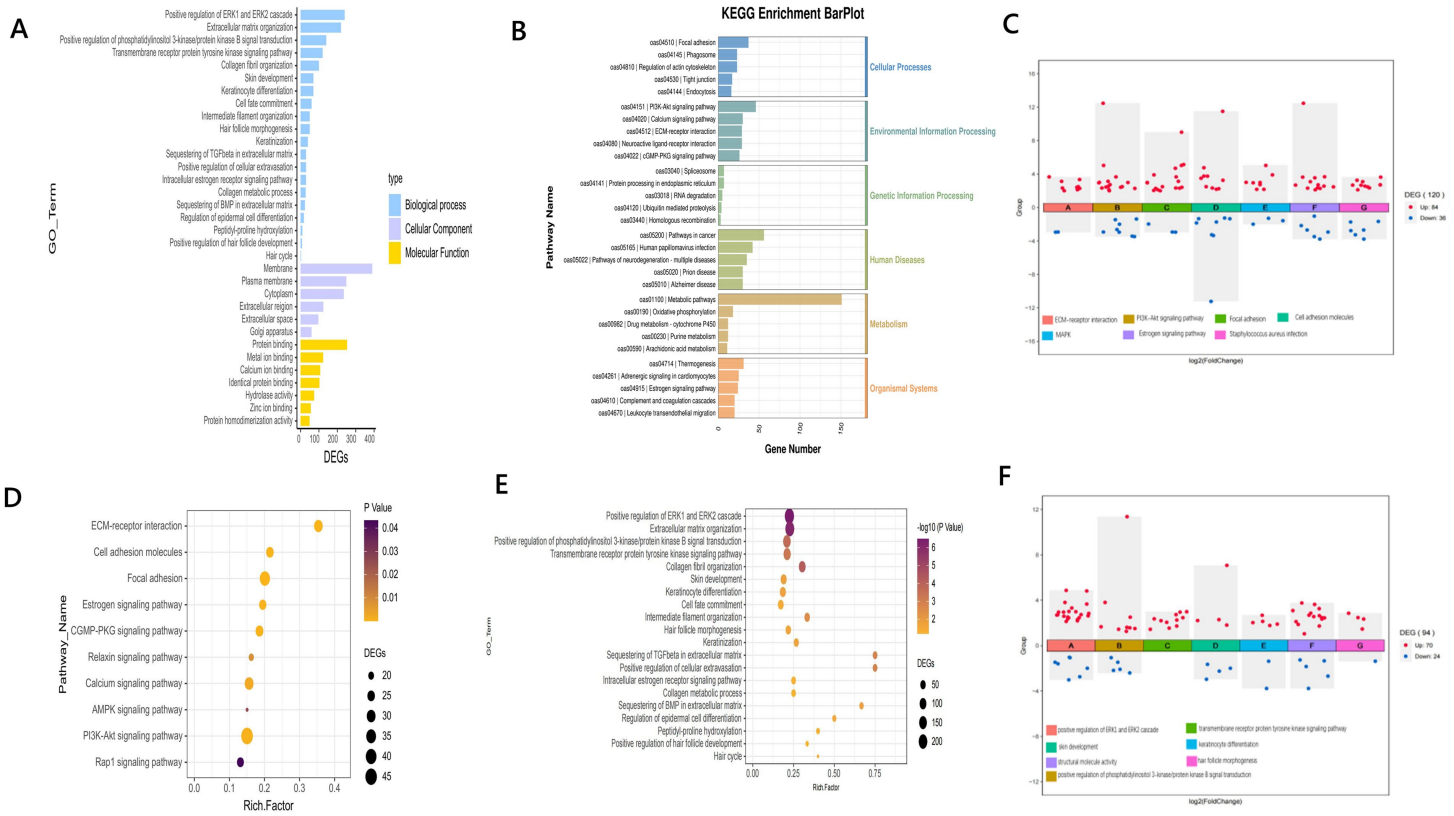
**(A)** GO enrichment bar plot; **(B)** KEGG enrichment bar plot; **(C)** DEGs in the KEGG pathway related to hair follicle development; **(D)** KEGG enrichment scatter plot; **(E)** GO en-richment scatter plot; **(F)** DEGs in the GO pathway related to hair follicle development.

To further analyze the functions of differentially expressed genes (DEGs) in the skin tissues of Qianhua Mutton Merino sheep at different ages, enrichment analysis of DEGs was conducted using the DAVID software product (Table 3). The results of the GO enrichment analysis showed (Figure 4A) that in Biological Processes (BPs), the most significantly enriched terms were the positive regulation of the ERK1 and ERK2 cascade (GO:0070374) and PI3K-Akt (GO:0051897). In Cellular Components (CCs), the most significantly enriched terms were membrane (GO:0016020), plasma membrane (GO:0005886), and cytoplasm (GO:0005829). In Molecular Function (MF), the most abundant terms were protein binding (GO:0005515) and metal ion binding (GO:0046872). Enrichment analysis revealed that several differentially expressed genes (DEGs) were enriched in entries related to hair follicle development (Figures 4C,F), such as structural molecule activity, hair follicle morphogenesis (GO:0031069), keratinocyte differentiation (GO:0030216), keratinization (GO:0031424), and skin development (GO:0043588). These functions are crucial for the formation, maturation, and establishment of the stratum corneum of hair follicles. The enrichment of the hair cycle (GO:0042633) suggests that some DEGs may be involved in the dynamic regulation of the hair anagen (growth) phase, catagen (regression) phase, and telogen (resting) phase. In the context of structural molecule activity, the differentially expressed genes (DEGs) include *KRT18*, *KRT23*, *KRT25*, *KRT39*, *CRYAB*, *KRT27*, *KRT33B*, *KRT24-1*, *CLDN4*, *CLDN23*, and *CLDN8*. In the hair follicle morphogenesis pathway, the DEGs are *KRT71*, *KRT25*, *KRT27*, and *GLI2*. Within the PI3K-Akt pathway, the DEGs include *CCL21*, *IL18*, *LRP2*, *IGF-2*, *MFHAS1*, *STOX1*, *HB-EGF*, *NRG1*, and *CHI3L1*. The results of KEGG enrichment analysis (Figures 4B,D,E) showed that differentially expressed genes (DEGs) were significantly enriched in multiple key biological pathways. Among these pathways, the significantly enriched pathways include ECM–receptor interaction, the PI3K-Akt signaling pathway, focal adhesion, the estrogen signaling pathway, and the relaxin signaling pathway, indicating the role of hormonal regulation in hair follicle development. The enrichment of the MAPK signaling pathway and calcium signaling pathway plays a role in cellular energy metabolism, stress response, and other physio-logical processes. In the estrogen signaling pathway, the differentially expressed genes (DEGs) include *KRT18*, *MMP2*, *ADCY2*, *KRT23*, *ESR1*, *CREB3L3*, *KRT25*, *KRT39*, *KRT26*, and *KRT27*. In the PI3K-Akt pathway, the DEGs are *VEGF-D*, *LAMA2*, *PDGFRA*, *CREB3L4*, *ITGA8*, *COL4A3*, *ITGA9*, *TNXB*, *FLT-4*, *CREB3L3*, and *FGF21*. In the MAPK signaling pathway, the DEGs consist of *VEGF-D*, *PDGFRA*, *CSF1R*, *FLT-4*, *DUSP10*, *MAPK23*, *CD14*, *FGF21*, *MAPK12*, and *MAPK13*.

**Table 3:** Kegg enrichment analysis of DEGs.

Pathway name	DEGs
ECM-receptor interaction signaling pathway	*LAMA2, ITGA8, COL4A3, ITGA9, TNXB, LAMC3, PG4, α4(IV) chain, α6(IV) chain, LAMA5, LAMA11, LAMC1, α3(IV) chain, HSPG2, TSP1, α1(IV) chain, α2(IV) chain, LAMB1, THBS3, FN1, ITGB3, CD36, ITGA4, LAMB2, ITGA10, RELN, TSP2*
PI3K-Akt signaling pathway	*VEGF-D, LAMA2, PDGFRA, CREB3L4, ITGA8, COL4A3, ITGA9, TNXB, LAMC3, CSF1R, α4(IV) chain, GHR, FLT – 4, CREB3L3, PIK3R6, α6(IV) chain, LAMA5, LAMA11, LAMC1, FGF21, IL-6R, α3(IV) chain, Cyclin E1, NR4A1, MYB, TSP1, α1(IV) chain, α2(IV) chain, LAMB1, THBS3, FN1, ITGB3, ITGA4, LAMB2, PDGFRβ, ITGA10, RELN, LPAR1, PP2A Bγ, GYS-1, TSP2, IL7, erbB – 4, CREB3L2*
Focal adhesion signaling pathway	*VEGF-D, LAMA2, PDGFRA, MYLPF, ITGA8, COL4A3, ITGA9, TNXB, FYN, LAMC3, α2(IV) chain, FLT – 4, PIPKβ, MLCK2, α6(IV) chain, LAMA5, LAMA11, LAMC1, Cav-3, Shc2, α3(IV) chain, TSP1, α1(IV) chain, LAMB2, filamin C, THBS3, FN1, LAMB3, ITGA4, PDGFRβ, ITGA10, RELN, TSP2*
Cell adhesion molecules signaling pathway	*ITGA8, ITGA9, ITGAM, ALCAM, PCDH15, JAM3, Siglec – 1, VTCN1, CD34, ICAM1, VCAM – 1, HLA, SLA – DQA, NCAM2, CLDN4, CLDN 23, CLDN 8, CLDN 3, CLDN7, selectin L, ITGA4, CLDN 6, JAM2, CLDN 17*
MAPK signaling pathway	*VEGF-D, PDGFRA, CSF1R, FLT – 4, DUSP10, MAP2K3, CD14, FGF21, MNK1, RasGRP3, CACNA2D3, NR4A1, TGFβR2, GADD45γ, FLNC, PDGFRβ, MAPK12, VGCCs, MAPK13, RasGRP4, PLA2G4E, erbB – 4, STMN1, MEF2C*
Estrogen signaling pathway	*CREB3L4, KRT18, PLCβ1, MMP2, ADCY2, KRT23, ESR1, CREB3L3, KRT25, KRT39, KRT26, KRT27, SHC2, NESP55, KRT33B, HB – EGF, KRT40, KRT48 kDa,8C-1, KRT16, CaM, ADCY4, K33, V15, CREB3L2*

### 3.6 STRING protein–protein interaction analysis

**Figure 5:**
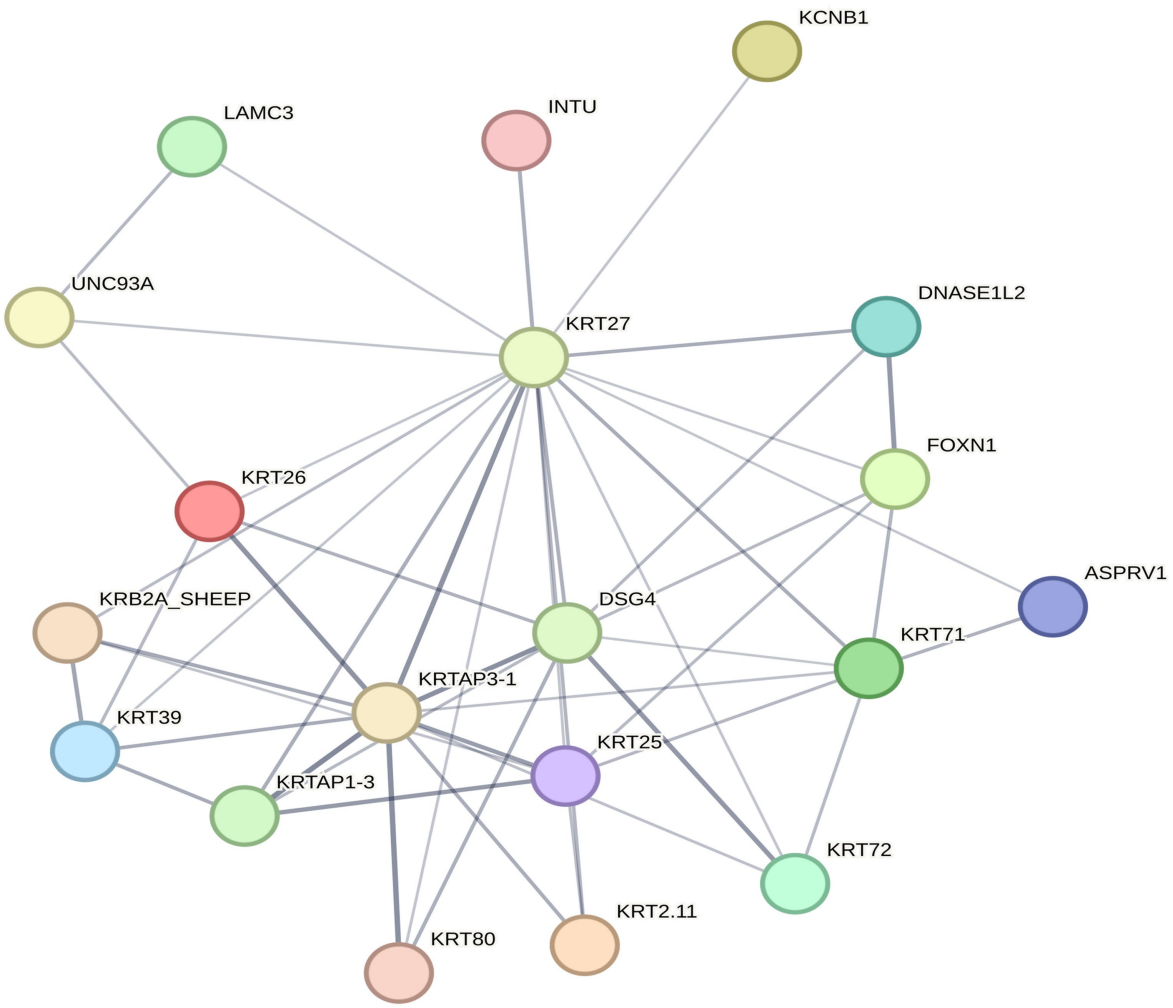
STRING protein–protein interaction analysis.

Through STRING protein–protein interaction network analysis (Figure 5), it was found that the *KRT27* gene serves as a core node among differentially expressed genes related to hair follicle development, closely interacting with several keratin genes as-sociated with hair follicle development, such as *KRT26*, *KRT*7*1*, *KRT39*, and *KRT25*. Moreover, the *KRT27* gene is significantly enriched in the estrogen signaling pathway, as well as in the entries for structural molecule activity and hair follicle morphogenesis, indicating that it may play an important role in hair follicle development.

### 3.7 Detection of the expression level of the KRT27 gene in hair follicle tissues at different ages

**Figure 6:**
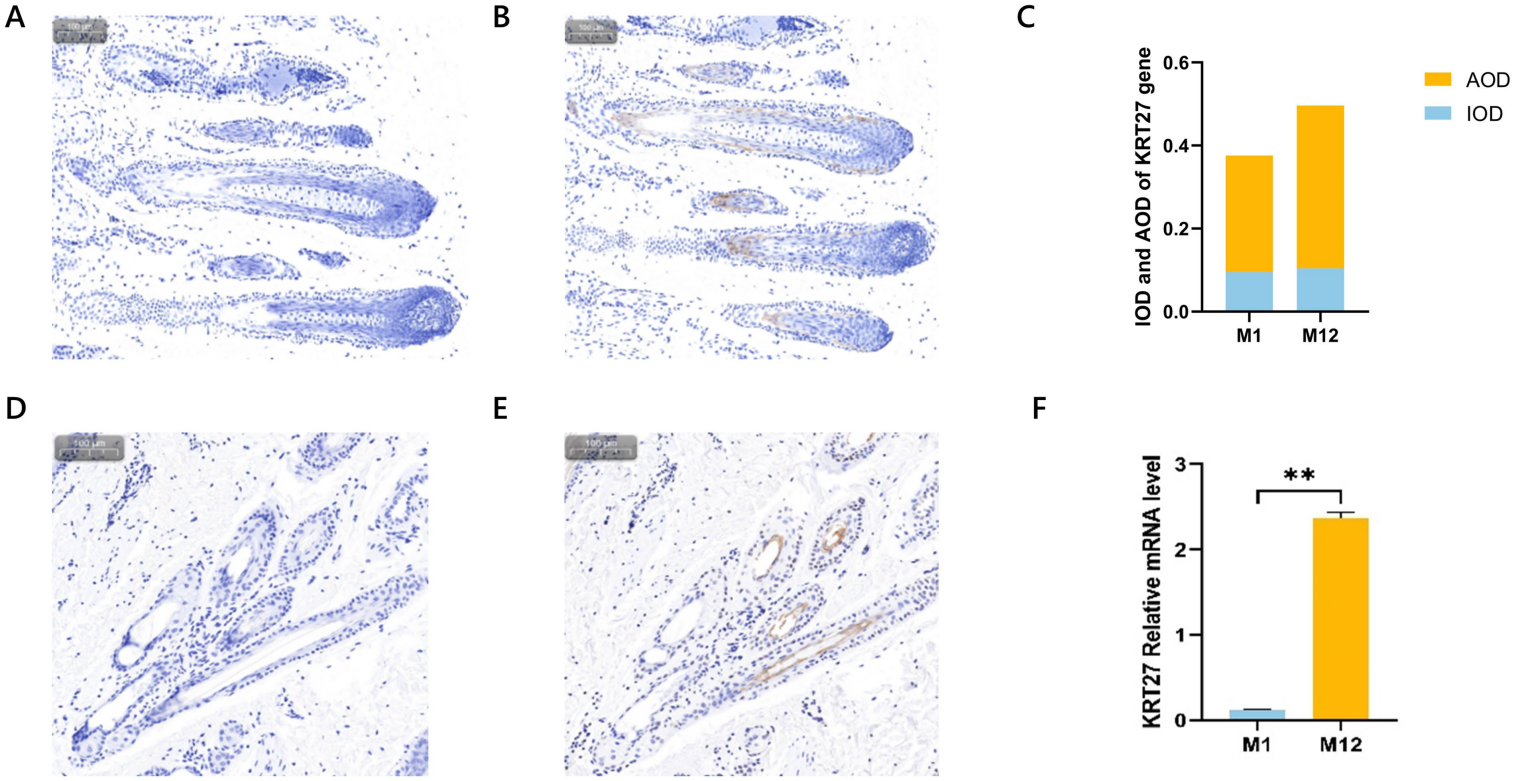
The tissue expression levels of *KRT27* in the skin hair follicles of sheep of different ages. **(A)** The control group of longitudinal sections of newborn skin tissue; **(B)** the experimental group of longitudinal sections of newborn skin tissue; **(C)** IOD and AOD of the *KRT27* gene; **(D)** the one-year-old group of longitudinal sections of newborn skin tissue; **(E)** the experimental group of longitudinal sections of newborn skin tissue; **(F)** RT-qPCR.

In this experiment, we employed immunohistochemistry (IHC) methods to ex-amine the skin tissues of newborn and one-year-old Qianhua Mutton Merino, aiming to investigate the tissue localization of *KRT27* in the hair follicles of Qianhua Mutton Merino at different ages (Figures 6A,B,D,E). The hair follicle structures in the M1 group were relatively closely arranged, with clear structures and a more regular morphology, indicating that they were in the stage of incomplete maturation. In contrast, the hair follicles in the M12 group exhibited a more complex morphology, with some curvature and branching and a relatively loose arrangement of cells, indicating that the hair follicles had fully matured. After DAB staining of the sections (Figures 6B,E), it was observed that the *KRT27* positive expression areas were brownish-yellow, a trait specifically expressed in the inner root sheath of the hair follicles. This finding indicates that *KRT27* is localized in the inner root sheath region of the skin hair follicles of both newborn and one-year-old Qianhua Mutton Merino.

The results of the RT-qPCR validation (Figure 6F) indicate that *KRT27* is expressed in both tissue groups, with a lower expression level in the M1 group compared to the M12 group, and the M12 group’s expression level was significantly upregulated (*p* < 0.01).

The analysis of the average optical density and cumulative optical density values from immunohistochemistry revealed that the expression level of the *KRT27* gene in M12 is higher than that in M1 (Figure 6C).

## 4 Discussion

### 4.1 The tissue structure of the skin hair follicles of Qianhua Mutton Merino sheep

Skin hair follicles are where wool is produced in wool-bearing sheep. As one of the few periodically growing micro-organs in a sheep’s body, a hair follicle relies on its cyclical growth to facilitate the growth and shedding of wool (13). Therefore, the characteristics and tissue structure of the hair follicle determine the quality and yield of wool in wool-producing animals. The skin hair follicles of fine wool sheep can be classified into primary hair follicles and secondary hair follicles based on their developmental timing and tissue structure (14). Primary hair follicles develop between 50 and 60 days of gestation and complete their development by the time of birth. These follicles are characterized by larger hair bulbs and longer and thicker follicles, and they possess two well-developed sebaceous glands. The hair shafts produced typically contain a medulla. In contrast, secondary hair follicles appear between 80 and 90 days of gestation. They are characterized by smaller hair bulbs and shorter and finer follicles, and they either lack or possess only one underdeveloped sebaceous gland. The hair shafts from these follicles do not contain a medulla (15–17). In the follicular characteristics of fine-wool sheep, the S/*p* value (the ratio of secondary follicles to primary follicles) is a trait with moderate heritability (0.3–0.4), being minimally influenced by non-genetic factors. It is one of the important indicators reflecting the genetic characteristics of sheep breeds and is closely related to the yield and quality of wool (18, 19). In their study of semi-fine hair in Yunnan, Hong Qionghua et al. found that the number of primary hair follicles at birth and at 1 year old was 10.50/mm ^2^ and 3.55/mm ^2^, respectively; the number of secondary hair follicles was 36.94/mm ^2^ and 26.26/mm ^2^, respectively; and the S/P ratios were 3.52 and 7.39, respectively (20). The number of primary hair follicles of cashmere goats at birth and in adulthood was 9.97/mm^2^ and 4.01/mm^2^, respectively; the number of secondary hair follicles was 60.75/mm^2^ at birth and 46.83/mm^2^ in adulthood; and the S/P ratio at birth was 6.10, while the S/P ratio in adulthood was 11.82 (21). This study found that, at birth, Qianhua Mutton Merino sheep had an average of 8.15 ± 1.04 primary hair follicles/mm^2^ and 61.81 ± 3.45 secondary hair follicles/mm^2^, with an S/P ratio of 7.58 ± 0.64. By 1 year of age, these values changed to 4.38 ± 5.21 primary hair follicles/mm^2^, 55.31 ± 5.21 secondary hair follicles/mm^2^, and an S/P ratio of 12.64 ± 1.02. Compared to fine-wool sheep and wool-producing fine-wool sheep, the number of primary hair follicles in Qianhua Mutton Merino sheep at birth is lower than that of the corresponding breeds both domestically and internationally, while the number of secondary hair follicles and the S/P ratio are higher than those of the corresponding breeds. At 1 year of age, the number of primary hair follicles is higher than that of the corresponding breeds both domestically and internationally, and the number of secondary hair follicles and the S/P ratio are also higher than those of the corresponding breeds. It is speculated that this may be due to the Qianhua Mutton Merino sheep being a dual-purpose breed for meat and wool, with a tendency toward meat production and relatively rapid body development, resulting in an increased body surface area. Consequently, the density of hair follicles is lower than that of breeds primarily used for wool or those that are dual-purpose for both wool and meat.

### 4.2 The genes and signaling pathways related to the development of skin hair follicles in Qianhua Mutton Merino sheep

Hair follicle formation is jointly regulated by a series of related factors and signaling pathways. There has been considerable research related to hair follicle development, and it has been found that various molecular signaling pathways, including Wnt, BMP, TGF-*β*, PI3K-Akt, and MAPK, are involved in this process (22). In addition, with the continuous development of high-throughput sequencing technology, numerous studies have indicated that various non-coding RNAs, such as lncRNA, miRNA, and circRNA, are also involved in the process of hair follicle development (23–25). Different signaling pathways, non-coding RNAs, and other important regulatory factors form a complex molecular regulatory network that jointly regulates the development of hair follicles. Among various signaling pathways, the PI3K-Akt signaling pathway plays a crucial role in the development of hair follicles by promoting communication between epidermal and dermal cells (26). The PI3K-Akt signaling pathway can activate the Wnt signaling pathway by increasing the phosphorylation levels of β-catenin and inhibiting the phosphorylation of glycogen synthase kinase-3b, thereby promoting the growth of hair follicles (27). Xiaoyang Lv et al. found that many genes, such as *FGF12* and *ATP1B4*, are enriched in the PI3K-Akt signaling pathway related to hair follicle growth and development (28). In this study, it was also found that 14 DEGs were enriched in the PI3K-Akt signaling pathway. The trend of changes in these genes may be closely related to the development and growth of hair follicles. MAPK is another important signaling pathway in regulating hair follicle development. It contributes to the formation of the hair follicle substrate and the cyclical growth of hair follicles by participating in the mechanisms of regulating apoptosis, differentiation, proliferation, and stress (29). In this study, it was found that 24 DEGs were enriched in MAPK, indicating that the MAPK signaling pathway is involved in the development of skin hair follicles in Qianhua Mutton Merino sheep.

This study found that the secondary hair follicles of Qianhua Mutton Merino sheep are still in the process of development at birth. There are extremely significant differences between birth and age in terms of the number of secondary hair follicles, the diameter and depth of the hair follicles, and the diameter of the wool fibers growing from the secondary hair follicles. It can be seen that secondary hair follicles are still in dynamic development after birth. In this study, DEGs related to hair follicle development were identified as IGF-2, FGF21, VEGF-D, and KRT16, KRT25, KRT26, KRT27, KRT33, and KRT39 of the KRT family. The above-mentioned genes are enriched in hair follicle-development-related signaling pathways such as PI3K-Akt, MAPK and Estrogen. Fibroblast growth factor 21 (*FGF21*) is a key regulatory factor in hair follicle development and cycling (30). Liu et al. found that *FGF21* induces AKT expression, subsequently leading to the AKT-mediated phosphorylation of target molecules, thereby influencing the intricate signaling network that regulates hair growth (31). Basharat Bhat et al. found that insulin-like growth factor 2 (*IGF2*) promotes hair follicle development during the growth phase of goats through MAPK signaling (32). Vascular endothelial growth factor (*VEGF*) plays a key role in promoting angiogenesis and supporting hair follicle growth (33). Keratin, a member of the intermediate filament superfamily, is the primary structural component of hair and hair follicles. The specific types and quantities of keratin not only determine hair characteristics but are also vital for maintaining its structural stability (34). Wen et al. identified 57 differentially abundant proteins (DAPs) in the skin of goat necks through multi-omics studies. These proteins from the keratin family are highly expressed during both the growth and regression phases (35). Therefore, the keratin family plays a role in determining the morphology of hair follicles and fibers. Research by Yu et al. indicates that the asymmetric expression of the *KRT27* gene is associated with hair whorl deviation and follicle curvature, playing a role in the de-termination of hair follicle morphology (36). Haoran Sun et al. discovered that lncRNA targets the estrogen signaling pathway and the PI3K-Akt signaling pathway through the *KRT27* gene in Dorper sheep (10). As a type I keratin gene, the expression level of KRT27 in different sheep breeds is significantly related to the distribution density of hair follicles in specific areas (37). Therefore, it was inferred that the *KRT27* gene could serve as a candidate for exerting regulatory functions.

## 5 Conclusion

The number of primary and secondary hair follicles in Qianhua Mutton Merino sheep at birth was significantly higher than that at the age of one, while the opposite was true for the S/P value. Qianhua Mutton Merino DEGs in the skin tissues of newborn and one-year-old sheep, including *IGF-2*, *FGF21*, and *VEGF-D*, as well as *KRT16*, *KRT25*, *KRT26*, *KRT27*, *KRT33*, and *KRT39* of the KRT family, are significantly enriched in signaling pathways related to hair follicle development, such as the PI3K-Akt, MAPK, and Estrogen signaling pathways, indicating that the above mentioned signaling pathways are involved in hair follicle development.

## Data Availability

The original contributions presented in the study are publicly available. This data can be found here: The data has been uploaded to the SRA of NCBI, Link: http://www.ncbi.nlm.nih.gov/bioproject/1366756.

## Footnotes

1
https://bioconductor.org/packages/release/bioc/html/edgeR.html

2
https://cn.string-db.org/

3
https://david.ncifcrf.gov/
